# An investigation of the added value of an ACPA multiplex assay in an early rheumatoid arthritis setting

**DOI:** 10.1186/s13075-015-0786-z

**Published:** 2015-10-05

**Authors:** Jurgen van Heemst, Leendert A. Trouw, Leonor Nogueira, Hanna W. van Steenbergen, Annette H. M. van der Helm-van Mil, Cornelia F. Allaart, Guy Serre, Rikard Holmdahl, Tom W. J. Huizinga, René E. M. Toes, Diane van der Woude

**Affiliations:** Department of Rheumatology C1-R, Leiden University Medical Center, Albinusdreef 2, PO Box 9600, Leiden, 2300 RC The Netherlands; Laboratory of Epidermis differentiation and Rheumatoid Autoimmunity, Centre National de la Recherché Scientifique, Avenue de Grande-Bretagne 330, TSA 40031, Toulouse, 31059 Cedex 9 France; Department of Medical Biochemistry and Biophysics, Medical Inflammation Research Unit, Karolinska Institutet, Stockholm, SE-171 77 Sweden

## Abstract

**Introduction:**

Recently, arrays have become available that allow the simultaneous analysis of several anti-citrullinated protein antibody (ACPA) reactivities using distinct citrullinated peptides. Such assays are designed for exploratory studies. The interpretation of positive antibody reactivities can best be made if the diagnostic and prognostic value of a multiplex array in an early arthritis setting is known and if the multiplex-positive patients who are negative according to three commonly used commercial ACPA assays are characterized.

**Methods:**

Using Thermo Scientific’s ImmunoCap ISAC (Immuno Solid-phase Allergen Chip) system, a multiplexed array that determines reactivities to 11 citrullinated peptides, we analysed serum/plasma of 195 healthy controls and 1282 early arthritis patients from two independent cohorts: the Leiden Early Arthritis Clinic (n = 1013) and the IMPROVED (n = 269) cohort. Findings were compared with results primarily of the anti-citrullinated cyclic peptide 2 (anti-CCP-2) assay but also with anti- CCP-3 and anti-mutated citrullinated vimentin (anti-MCV) assays. The associations between ACPA reactivities and patient characteristics, risk factors (shared epitope, smoking) and disease outcomes (progression of undifferentiated arthritis to rheumatoid arthritis (RA) and severity of joint destruction) were assessed.

**Results:**

Thirty-one percent of anti-CCP-2-negative RA patients displayed reactivity toward citrullinated peptides in the multiplex assay. These patients had a positive signal toward a more restricted peptide repertoire than anti-CCP-2-positive RA patients (median of 1 versus 5). Within anti-CCP-2-negative patients, ACPA reactivity as detected by multiplex array was not significantly associated with known risk factors or clinical or prognostic parameters. The frequency of sera from anti-CCP-2-negative RA patients who were positive for the multiplexed peptides was comparable to the frequency in non-RA arthritic patients (27 %).

**Conclusions:**

Additive citrulline peptide reactivities detected by the current multiplex system did not reach significant power to be RA-specific. The presence of residual citrulline reactivities detected by this multiplex system in arthritis patients who are negative in commercial ACPA assays needs to be interpreted with caution.

**Electronic supplementary material:**

The online version of this article (doi:10.1186/s13075-015-0786-z) contains supplementary material, which is available to authorized users.

## Introduction

Rheumatoid arthritis (RA) is a systemic inflammatory disease characterized by extensive inflammation of synovial joints [1]. Patients with RA frequently present with autoantibodies, of which the anti-citrullinated protein antibodies (ACPAs) are of particular interest. These autoantibodies are highly specific for RA and specifically target proteins that have undergone a post-translational modification that converts arginine to citrulline residues. ACPAs can be used to predict the development of RA as these autoantibodies can be detected up to 10 years prior to disease onset [2, 3]. Furthermore, ACPAs can subdivide patients with RA in distinct disease subsets. These subsets differ substantially in clinical outcome and with regard to underlying genetic and environmental risk factors [4–6].

Several commercial tests are available that use specific citrullinated antigens to determine ACPA positivity. The most widely used tests are anti-citrullinated cyclic peptide 2 (anti-CCP-2), anti-CCP-3 and anti-mutated citrullinated vimentin (anti-MCV) [7, 8]. Anti-CCP-2 has been used in most studies and has long been the standard test to determine reactivity to citrullinated peptides, although the sequence identity has not been disclosed [9]. It has been postulated that because commercial anti-CCP-tests employ peptide sequences that are most likely not derived from human proteins, these tests do not detect part of the citrullinated-peptide reactivities [10]. Therefore, arrays have been developed to do exploratory studies on a large number of peptides. This could be important to enhance understanding of disease as well as identify antibody systems that are predictive of disease characteristics which can occur in a number of different diseases such as exocrine gland dysfunction, which is associated with anti-Sjögren’s syndrome-related antigen A (anti-SSA) antibodies.

Recently, arrays have become available that can determine citrullinated peptide reactivities toward a wide variety of natural citrullinated antigens in a relatively high-throughput setting [10, 11]. Intriguingly, these arrays showed the presence of reactivities against citrullinated peptides within anti-CCP-2-negative RA patients. This raised the question of what a positive signal in such an array means, and to guide subsequent exploratory studies, we determined whether a positive signal in a multiplex assay has an additive value over commercial assays determining the presence of ACPA and whether these patients share the characteristics of RA patients positive in commercial ACPA assays.

We aimed to perform a detailed characterization of the value of multiplex ACPA testing in an early arthritis setting by studying the characteristics of anti-CCP-2-negative patients who are positive or negative for additional citrulline reactivities. We studied clinical parameters and the presence of genetic and environmental risk factors by using a previously validated multiplex chip-based array, the ImmunoCAP ISAC (Immuno Solid-phase Alergen Chip) system [11–14]. Using this array, we measured reactivity against 11 different citrullinated peptides derived from fillagrin, type II collagen, alpha-enolase, fibrinogen and vimentin in patients from two independent arthritis cohorts.

## Methods

### Patients

Citrullinated peptide reactivities were assessed on baseline plasma samples of arthritis patients (n = 1013) included in the Leiden Early Arthritis Clinic (EAC). This Dutch cohort was initiated in 1993 and includes patients with a recent onset of arthritis (symptom duration of less than 2 years) from the Leiden area [15, 16]. Definitive diagnoses were determined after 1 year of follow-up by an experienced rheumatologist. In the current analysis, 564 patients fulfilled the American College of Rheumatology (ACR) 1987 criteria for RA and they are called ‘Leiden RA patients’. Patients not fulfilling the RA criteria, who had no other definite clinical diagnosis, were classified as undifferentiated/unclassified arthritis (UA) (n = 297). The remaining 149 patients were classified as non-UA/non-RA patients; 135 out of 149 patients were anti-CCP-2-negative and these presented with a wide variety of diagnoses (Additional file 1).

We also assessed citrullinated peptide reactivities in baseline serum samples of 269 patients who were included in the IMPROVED study and who fulfilled the ACR 1987 criteria for RA at the time of inclusion [17]. The IMPROVED study (ISRCTN11916566) was a randomized controlled trial investigating the effect of different treatment regimens in early arthritis.

Genotyping of the HLA-DRB1 region in order to determine the number of RA-associated shared epitope (SE) alleles was available for 500 patients (EAC only) [18]. Within the EAC, radiographs were taken at baseline and at yearly follow-up visits for 7 years. In total, 2340 sets of hands and feet radiographs of 463 patients with RA were scored by using the Sharp-van der Heijde scoring (SHS) methods by one experienced reader with known time order (intra-class correlation coefficient of 0.91) [19].

Patient samples were compared with serum samples of 195 healthy controls. These controls lived in the Leiden area and were matched to the patients for age and gender. Controls were questioned extensively about the presence of rheumatic conditions and joint complaints and were excluded if such complaints were present. The prevalence of IgM-rheumatoid factor (IgM-RF) and anti-CCP-2 are comparable to those of the general population.

The study was performed in accordance with the Declaration of Helsinki, and protocols were approved by the Leiden University Medical Center ethics committee. Informed consent was obtained.

### Citrullinated peptide assays

Anti-CCP-2 was measured on the Immunoscan RA Mark 2 (Eurodiagnostica, Arnhem, The Netherlands) with a cutoff of 25 arbitrary units in all patients. Anti-CCP-3 and anti-MCV were measured in 454 EAC RA patients by, respectively, Quanta lite CCP 3.1 IgG/IgA (Inova Diagnostics Inc., San Diego, CA, USA) and Orgentec Diagnostika GmbH (Mainz, Germany) with a cutoff of 20 arbitrary units. Cutoffs were set by using the instructions of the manufacturer. IgM-RF was determined by an enzyme-linked immunosorbent assay (ELISA)-based system with a cutoff as specified by the manufacturer in 614 patients with RA (EAC and IMPROVED cohort).

Antibody responses to citrullinated peptides were measured with the ImmunoCAP ISAC system (Thermo Fisher Scientific) that contains 11 different citrullinated peptides and their arginine counterparts: CCP1, CitC1 (CII355-378cit3), CEP1, Fibα36-50, Fibα621-635, Fibβ36-52, Fibβ60-74, Fibβ563-583, Fibβ580-600, Vim2-17 and Vim60-75 as described elsewhere [11–14, 20]. We excluded 4 % of the tested samples from the analyses as antibody responses could not be accurately determined because of high background signals or unspecific reactivity toward streptavidin.

For cutoff calculations, the citrulline-specific signal was used (difference in fluorescence intensity between the citrullinated peptide and the arginine-containing peptide). The cutoff was established as the mean plus two times the standard deviation (SD) of the citrulline-specific signals in 195 healthy control sera. For CitC1 (a peptide derived from collagen type II), we used the citrulline signal without subtracting the arginine signal as this is a non-linear peptide and has independent but exclusive reactivities to the arginine counterpart [21].

For reasons of sample availability, plasma of EAC patients and serum of IMPROVED patients and healthy controls were used for the multiplex assay. For several individuals (n = 59), both serum and plasma was available. Results were comparable between serum and plasma in 92 % of cases (data not shown).

### Statistical analysis

Linear regression was performed to study the association between log-transformed median anti-CCP-2 levels and the number of peptides recognized in the multiplex array. Differences in anti-CCP-2 levels between groups were analysed with the Mann-Whitney test.

To determine the association between the risk factors of HLA-SE, smoking and RA, unconditional logistic regression analysis was performed to calculate odds ratios (ORs) and 95 % confidence intervals (CIs). Multiplex-negative RA patients (either anti-CCP-2-negative or -positive) were used as the reference category and compared with multiplex-positive RA patients (again in both categories: anti-CCP-2-positive or anti-CCP-2-negative). Similarly, the association between multiplex positivity or anti-CCP-2 positivity and progression to RA was assessed. To study progression, we used selected patients who are classified as UA patients at baseline and we assessed the development of RA in the first year of follow-up by using the ACR 1987 criteria for RA.

To study biological interaction between smoking, HLA-SE and multiplex positivity in anti-CCP-2-negative RA patients, logistic regression analysis was performed as described elsewhere [22]. To analyse the rate of joint destruction, a multivariate normal regression model for longitudinal data with log-transformed SHS as response variable was used as previously described [23]. This method analyses all repeated measures at once and takes advantage of the correlation between these measurements. Analyses were adjusted for age, gender and different inclusion periods as proxy for treatment strategy [15]. Anti-CCP-2-negative RA patients were used as reference and compared with anti-CCP-2-positive RA patients. We also used anti-CCP-2-negative multiplex-negative and anti-CCP-2-negative multiplex-positive RA patients as a reference and these were compared with, respectively, anti-CCP-2-negative multiplex-positive and anti-CCP-2-positive multiplex-positive RA patients. The beta (β) indicates the relative fold increase in radiographic joint damage per year.

## Results

### Presence of citrulline reactivities by multiplex assay in patients with RA

**Fig. 1:**
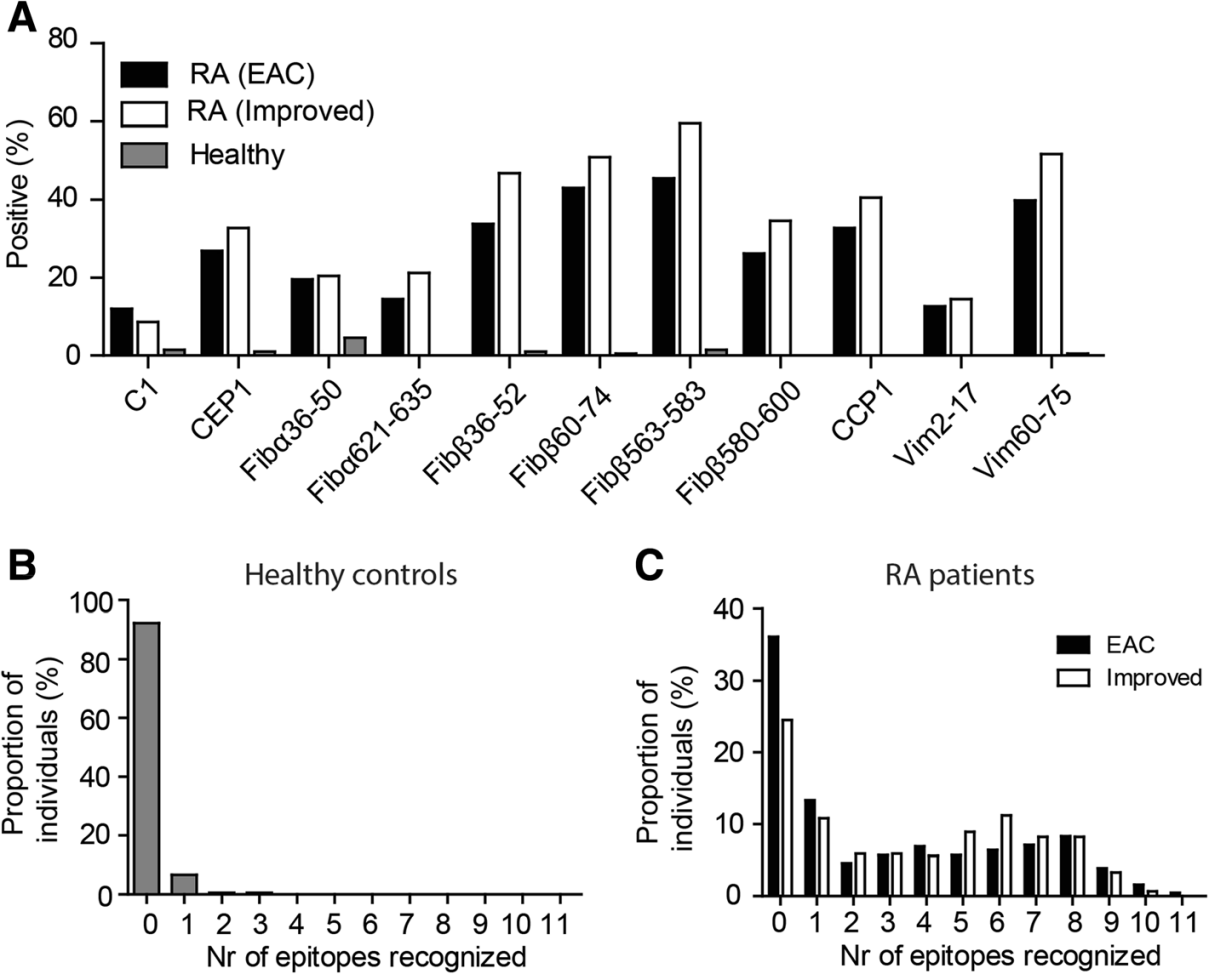
Citrulline reactivities in patients with RA and healthy controls. **a** Plot depicts the frequency of positive RA patients in the EAC (n = 564, in *black*) or the IMPROVED (n = 269, in *white*) cohort and healthy controls (n = 195, in *grey*) for the different tested citrullinated peptides. **b** Percentage of healthy controls plotted against the number of citrulline reactivities. **c** Percentage of patients with RA (EAC in *black* and IMPROVED in *white*) plotted against the number of citrulline reactivities. *CCP* cyclic citrullinated peptide, *EAC* Leiden Early Arthritis Clinic, *IMPROVED*, *RA* rheumatoid arthritis

Before embarking on determining the diagnostic value of a prototype multiplex array in an early arthritis setting, we wished to validate the assay by comparing the results in the Leiden population of patients with RA with previously published data by using other sets of patients with RA. We used a multiplex array to measure reactivities against the arginine and citrulline variants of 11 different antigens. A cohort of healthy controls was used to establish the cutoff for each of the tested antigens. All citrullinated peptides were recognized by a proportion of patients with RA in the EAC and the IMPROVED cohort (Fig. 1a). In 8 % of the tested healthy controls, citrulline reactivities were present and 13 out of 15 multiplex-positive controls recognized a single citrullinated peptide (Fig. 1b). In patients with RA, citrulline reactivities were observed in the majority of patients (64 % in EAC and 75 % in IMPROVED) and positive patients were generally positive for more than one peptide (79 % in EAC and 84 % in IMPROVED, Fig. 1c). Notably, the frequency of anti-CCP-2-positive RA patients is higher in the IMPROVED than in the EAC (65 % versus 51 %) and this is probably explained by the different inclusion criteria.

**Fig. 2:**
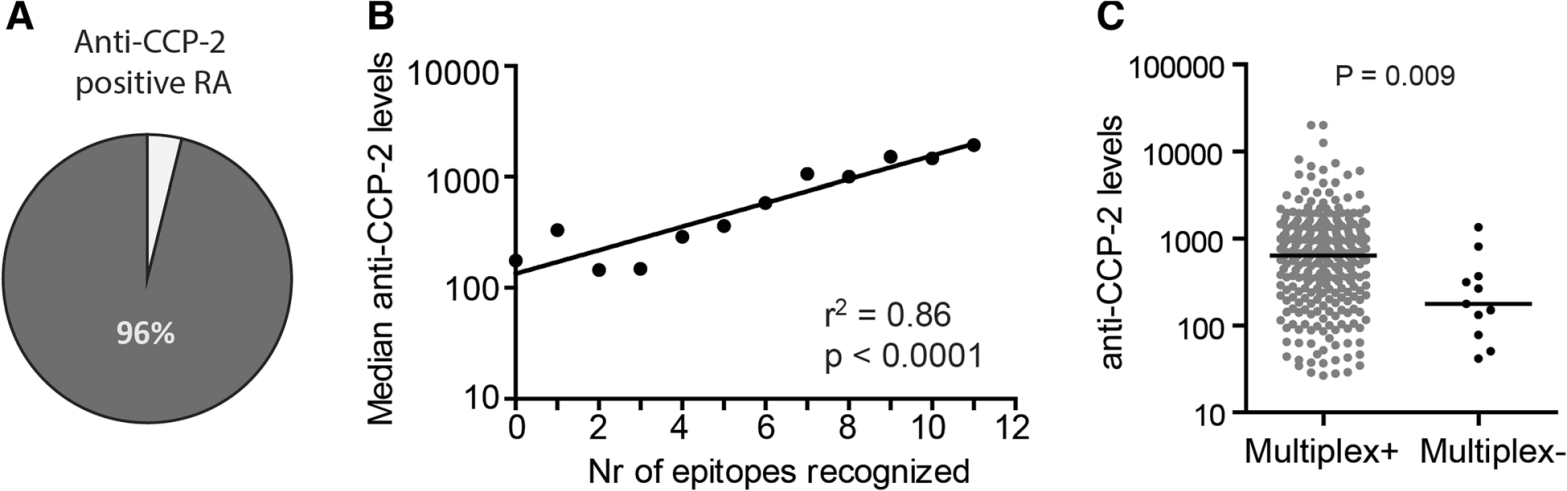
Multiplex positivity correlates with anti-CCP-2 levels. **a** Percentage of anti-CCP-2-positive RA patients (n = 285) in the EAC that are positive or negative in the multiplex array. **b** Median anti-CCP-2 levels plotted against the number of recognized peptides. A linear regression was used to determine the degree of correlation. **c** Anti-CCP-2 levels in multiplex-negative (n = 11) and -positive (n = 274) RA patients. Each dot represents an EAC patient. Median anti-CCP-2 levels were compared by using the Mann-Whitney test. *CCP* cyclic citrullinated peptide, *EAC* Leiden Early Arthritis Clinic, *RA* rheumatoid arthritis

We next focused on anti-CCP-2-positive RA patients. The multiplex chip-based array detected citrullinated peptide reactivities in 274 out of 285 (96 %) anti-CCP-2-positive EAC RA patients (Fig. 2a). Percentages were comparable in the IMPROVED cohort (169/174, 97 %) and with anti-MCV- or anti-CCP-3-positive EAC RA patients (241 out of 273 (88 %) and 234 out of 261 (90 %), respectively). The number of recognized citrullinated multiplex peptides associated with median anti-CCP-2 antibody levels (Fig. 2b, R^2^ = 0.86, *P* < 0.0001) as has also been reported for ACPA fine specificities determined by ELISA [24]. Likewise, the 4 % of anti-CCP-2-positive multiplex-negative RA patients displayed lower anti-CCP-2 levels (Fig. 2c, *P* = 0.009). These data show that nearly all anti-CCP/MCV-positive RA patients are positive in the multiplex array. The small multiplex-negative subgroup represents patients with lower anti-CCP-2 levels.

Most importantly, the test characteristics described above are comparable to previously published data, indicating that the multiplex array reproducibly detected reactivities against commonly used citrullinated peptide tests in patients with RA.

### Multiplex-positive anti-CCP-2-negative RA patients

**Fig. 3:**
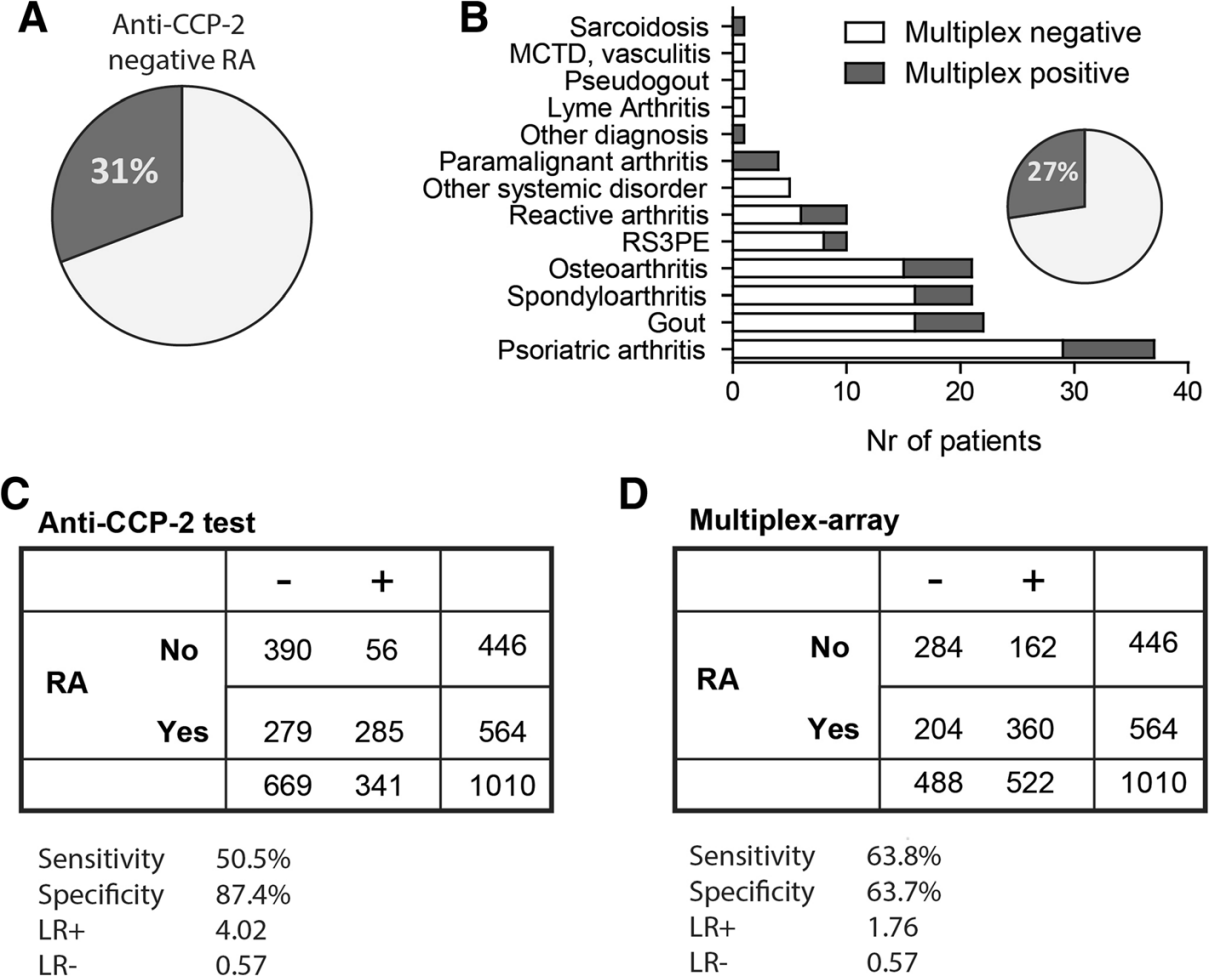
Multiplex positivity in anti-CCP-2-negative RA patients is not RA-specific. **a** Percentage of anti-CCP-2-negative RA patients (n = 279) in the EAC who are positive (n = 86) or negative (n = 193) in the multiplex array. **b** Pie diagram summarizes the percentage of multiplex-positive (n = 37, *grey*) and -negative (n = 98, *white*) anti-CCP-2-negative non-RA/UA patients (n = 135). Bar diagram plots the number of patients who are multiplex-positive and -negative with different diagnoses. **c**-**d** ACR 1987 criteria were used to calculate the sensitivity and specificity for RA of the anti-CCP-2 test (c) and of the multiplex array (d) by comparing patients who fulfilled the ACR 1987 criteria with patients who did not (UA patients or patients with a different form of arthritis). *ACR* American College of Rheumatology, *CCP* cyclic citrullinated peptide, *EAC* Leiden Early Arthritis Clinic, *LR* likelihood ratio, *MCTD* mixed connective-tissue disease, *RA* rheumatoid arthritis, *UA* undifferentiated arthritis

Recent reports using multiplex platforms with citrullinated peptides also showed positive response in anti-CCP-2-negative RA patients [10, 11, 24]. To determine whether this could also be found in the Leiden RA population, we next analysed the multiplex array reactivity in the anti-CCP-2-negative RA population. In the EAC, 31 % (86/279) of the anti-CCP-2-negative RA patients tested positive in the multiplex assay versus 34 % (31/91) in the IMPROVED cohort (Fig. 3a). Similar percentages were found in anti-CCP-3-negative (29 %, 56/193) or anti-MCV-negative (27 %, 49/181) RA patient populations. These findings are also comparable to previously reported observations using similar assays in other sets of patients. As anti-CCP-2-positive multiplex-negative RA patients are rare (4 %), it was not possible to perform detailed analyses in this population with sufficient power.

### Diagnostic value of the multiplex array in an early arthritis setting

The finding that a substantial proportion of ACPA-negative RA patients, as determined by three commercial assays, shows reactivity in the multiplex assay is intriguing and prompted us to determine whether this assay has an additive value over “conventional” ACPA testing in an “early arthritis setting”. We next analysed multiplex positivity in EAC patients with a form of arthritis other than UA or RA (so-called non-RA, n = 149). Thirty percent (45/149) of non-RA patients were multiplex-positive. In anti-CCP-2-negative (135/149) non-RA patients, 27 % (37/135) were multiplex-positive, without a predilection for a particular diagnosis (Fig. 3b). Similar findings were made when the anti-CCP-3 and anti-MCV assays were used to determine the presence of ACPAs.

The findings described above were reflected in the sensitivity and the specificity (in relation to the original ACR 1987 criteria) of the multiplex assay (63.8 % and 63.7 %) in comparison with the anti-CCP-2 ELISA (51 % and 87 %). To calculate the sensitivity and specificity, all 446 patients in the EAC without RA were taken into account. These patients had either UA or another form of arthritis. The positive likelihood ratio (LR^+^) for the multiplex is lower (1.76 versus 4.02 for anti-CCP-2), whereas the LR^−^ is similar (0.57) (Fig. 3c, d). These analyses were performed by using the ACR 1987 criteria for RA since the European League Against Rheumatism/ACR 2010 criteria include anti-CCP-2 status, thereby introducing potential circular reasoning.

The diagnosis of non-RA patients was initially assessed after 1 year by an experienced rheumatologist. To rule out the possibility that the multiplex-positive non-RA patients were misdiagnosed or that they developed RA later in time, we re-assessed their diagnosis, radiographs and medication use at their last visit. Two non-RA patients were now diagnosed with RA. When we took these findings into consideration and assumed that these two patients were initially misdiagnosed, the sensitivity of the multiplex assay increased from 63.7 % to 64.1 %.

All analyses described above have been performed by using the same cutoff (mean + 2 × SD of the citrulline-specific signal in healthy controls). However, using several different cutoffs did not change the outcome or conclusions of the studies (data not shown).

Together these data illustrate that a large subset of anti-CCP/MCV-negative arthritis patients is positive in the multiplex array. However, the multiplex has a low RA specificity.

### Characteristics of anti-CCP-2-negative RA patients positive or negative for citrullinated peptide reactivities

As the multiplex assay was designed to identify antibodies and antibody patterns to formulate new hypotheses regarding of value for understanding aetiology, pathogenesis, prognosis and prediction, we wished to investigate whether the multiplex assay could be useful to stratify anti-CCP-2-negative RA patients into different subsets using other outcome parameters. There were no statistical differences in baseline characteristics between multiplex-negative and -positive EAC patients, although female anti-CCP-2-negative patients were more often multiplex-positive (*P* = 0.04, Additional file 2). Given the large number of comparisons performed, this is not significant.

### Citrullinated-peptide repertoire recognized by anti-CCP-2-negative, multiplex-positive RA patients

We next analysed in more detail the reactivity patterns of sera from multiplex-positive anti-CCP-2-negative sera. Multiplex-positive anti-CCP-2-negative subjects (either RA or healthy or non-RA) recognized mostly one peptide (median of 1 in anti-CCP-2-negative versus 5 in anti-CCP-2-positive) as previously reported [11].

Eight-five percent of the anti-CCP-2-negative sera in the EAC that are positive for a single peptide are positive for Fibα36-50 or Fibβ563-583 (122/143) (Additional file 3). As the fraction of anti-CCP-2-negative multiplex-positive patients relies largely on these two peptide reactivities, we studied these in more detail (Additional file 4). Anti-CCP-2-negative patients with reactivities toward Fibβ563-583 were mostly positive just above the cutoff (Additional file 4). This was not unique for this peptide as this was also observed for other citrullinated peptide reactivities. Fibα36-50 was the exception as a substantial number of anti-CCP-2-negative RA patients tested clearly positive against this peptide (Additional file 4), although recognition of this peptide was not RA-specific. After removal of these two peptides from the analysis, 15 % of anti-CCP-2-negative RA patients were multiplex-positive versus 5 % of non-RA patients. This also led to a decreased sensitivity (54 %) but an increased specificity (81 %) of the multiplex assay.

### Risk factors and anti-CCP-2-negative RA patients positive or negative for citrullinated peptide reactivities

Previously, it was shown that the presence of anti-CCP-2 antibodies associates with HLA-SE alleles and smoking [5, 6]. Indeed, we found an increased frequency of HLA-SE alleles (43 % versus 79 %, OR = 4.82, *P* < 0.001) and smoking (22 % versus 32 %, OR = 1.57, *P* = 0.004) in anti-CCP-2-positive versus -negative RA patients (Table 1). In contrast, in the anti-CCP-2-negative subset, we found no difference in the frequency of HLA-SE alleles (46 % versus 39 %, OR = 0.74, *P* = 0.28) and smokers (21 % versus 21 %, OR = 0.90, *P* = 0.71, Table 1) between multiplex-negative and -positive RA patients. We did observe an increased frequency of HLA-SE alleles and smoking in multiplex-positive anti-CCP-2-positive RA patients compared with multiplex-positive anti-CCP-2-negative RA patients (79 % versus 38 % and 29 % versus 21 %, Table 1).

**Table 1:** Association of risk factors of HLA-SE and smoking with multiplex positivity in anti-CCP-2-negative RA patients

	Shared epitope			
	No, n (%)	Yes, n (%)	OR (95 % CI)	*P* value
CCP-2^−^	140 (57)	107 (43)	Reference	
CCP-2^+^	54 (21)	199 (79)	4.82 (3.26-7.14)	<0.001
CCP-2^−^Multiplex^−^	93 (55)	78 (46)	Reference	
CCP-2^−^Multiplex^+^	47 (60)	29 (39)	0.74 (0.42-1.28)	0.28
CCP-2^−^Multiplex^+^	47 (62)	29 (38)	Reference	
CCP-2^+^Multiplex^+^	51 (21)	192 (79)	6.10 (3.50-10.64)	<0.001
	Ever smoker			
	No, n (%)	Yes, n (%)	OR (95 % CI)	P value
CCP-2^−^	274 (78)	78 (22)	Reference	
CCP-2^+^	301 (69)	138 (31)	1.61 (1.17-2.22)	0.004
CCP-2^−^Multiplex^−^	183 (79)	54 (21)	Reference	
CCP-2^−^Multiplex^+^	90 (79)	24 (21)	0.90 (0.53-1.56)	0.71
CCP-2^−^Multiplex^+^	90 (79)	24 (21)	Reference	
CCP-2^+^Multiplex^+^	281 (71)	125 (29)	1.67 (1.01-2.74)	0.004

Statistical comparison of the frequency of smokers and HLA-SE alleles in anti-CCP-2-positive versus anti-CCP-2-negative RA patients and within multiplex-positive RA patients

*CCP* cyclic citrullinated peptide, *RA* rheumatoid arthritis, *OR* odds ratio, *CI* confidence interval

We also studied IgM-RF, which co-occurs with anti-CCP-2 (29 % in anti-CCP-2-negative and 91 % in anti-CCP-2-positive RA, *P* < 0.001), but again no increased frequency of IgM-RF was found in the subgroup of anti-CCP-2-negative multiplex-positive RA patients compared with multiplex-negative patients (25 % versus 33 %) [25]. These findings were replicated with anti-MCV- and anti-CCP-3 (data not shown). Together, these data show that anti-CCP/MCV-negative multiplex-positive RA patients cannot be distinguished from anti-CCP/MCV-negative multiplex-negative RA patients by using the read-outs described.

### Correlation of multiplex array positivity with clinical outcome in anti-CCP-2-negative patients

**Fig. 4:**
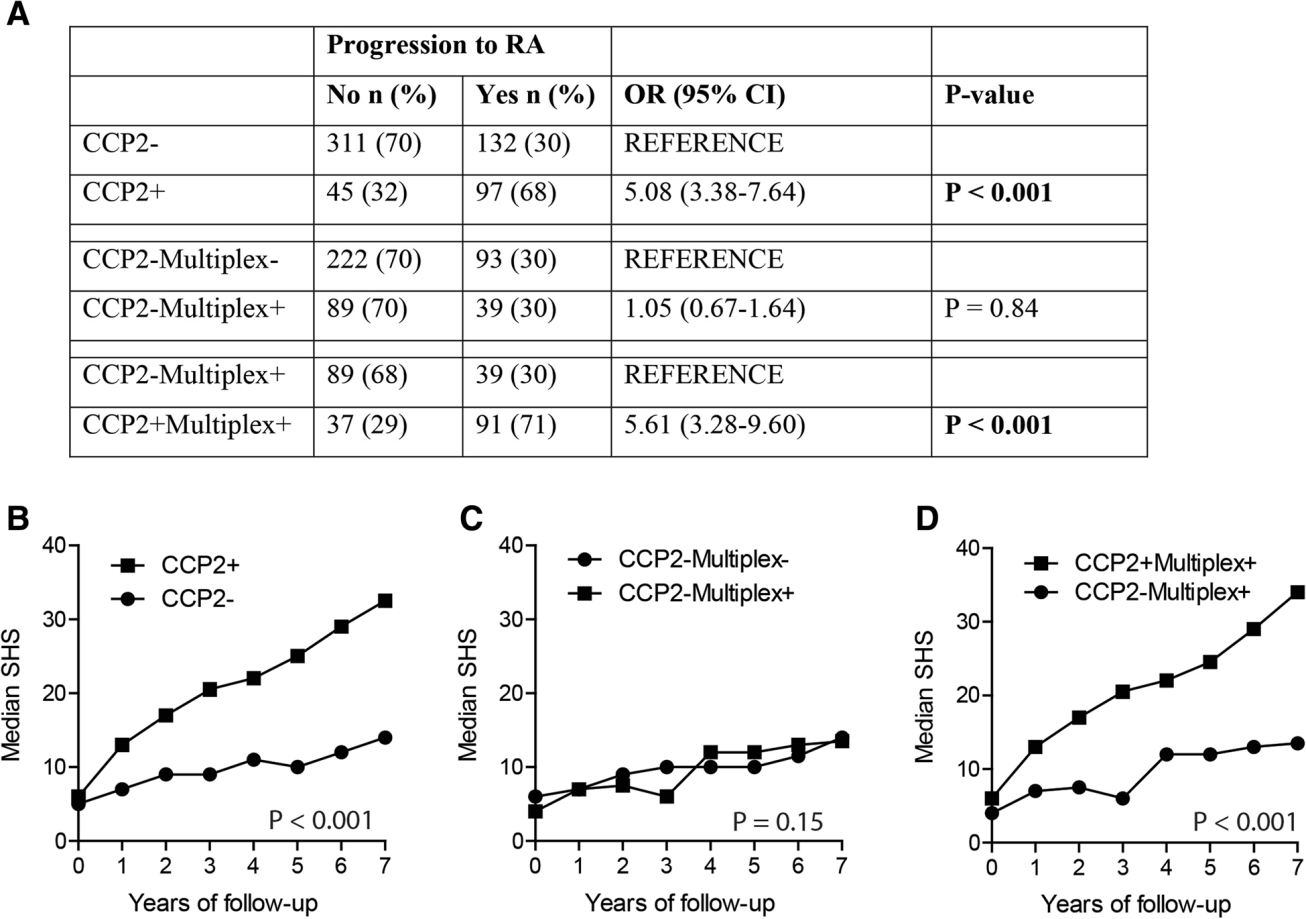
Multiplex positivity in anti-CCP-2-negative RA patients does not correlate with clinical outcome. **a** Scheme depicts the number of patients whose UA was diagnosed at baseline. Patients were followed for 1 year to study their progression toward RA. **b** Median SHS during 7 years of follow-up in anti-CCP-2-negative (n = 1010) and -positive (n = 1330) RA patients. **c** Median SHS during 7 years of follow-up in anti-CCP-2 negative RA patients who are multiplex-negative (n = 729) or -positive (n = 281). **d** Median SHS during 7 years of follow-up in multiplex-positive RA patients who are anti-CCP-2-negative (n = 281) or -positive (n = 1282). *CCP* cyclic citrullinated peptide, *CI* confidence interval, *N* number of radiographs, *OR* odds ratio, *RA* rheumatoid arthritis, *SHS* Sharp-van der Heijde score, *UA* undifferentiated arthritis

Next, we wished to study whether the multiplex assay aids in the prognosis of clinical outcome. In the EAC cohort, it is known that anti-CCP-2-positive UA patients have an increased risk to progress toward RA in the first year of follow-up as compared with anti-CCP-2-negative UA patients (30 % versus 68 %, Fig. 4a). Within the anti-CCP-2-negative subset, positivity in the multiplex array was not associated with progression (30 % in multiplex-negative versus 30 % in multiplex-positive). Accordingly, multiplex-positive UA patients who are anti-CCP-2-positive have a higher risk to progress than multiplex-positive anti-CCP-2-negative UA patients (71 % versus 30 %, Fig. 4a).

Likewise, with regard to joint damage, anti-CCP-2-positive RA patients had 11 % more joint destruction per year (β = 1.11, CI = 1.08-1.14, *P* < 0.001), which accumulated over 7 years of follow-up (Fig. 4b). There was no significant association with joint destruction over time when multiplex-negative anti-CCP-2-negative and multiplex-positive anti-CCP-2-negative patients were compared (β = 1.02, CI = 0.99-1.06, *P* = 0.15, Fig. 4c). In line with this observation, within the multiplex-positive RA subset, anti-CCP-2-positive patients have more joint destruction over time than anti-CCP-2-negative RA patients (β = 1.09, CI = 1.05-1.13, *P* < 0.001, Fig. 4d). Together, these data show no added clinical value of the multiplex array, using 11 citrullinated peptides, over the existing anti-CCP-2 test with regard to clinical phenotype or outcome of disease.

## Discussion

In this report, we extensively analysed the first multiplex array, consisting of 11 different citrullinated peptides for detection of ACPAs, in an early arthritis setting. The multiplex array can identify most RA patients who are positive in the conventional ACPA tests. A large subgroup of RA patients who are negative in commercial ACPA tests, including anti-CCP-2/3 and anti-MCV, are positive for one or more reactivities in the multiplex array. These reactivities have the potential to discover new autoantibody systems or lead to understanding of auto-immune reactions in RA. However, on a group level, these “additional” identified reactivities do not contribute additional clinical information. Furthermore, patients who are multiplex-positive but anti- CCP-2-negative differ from anti- CCP-2-positive RA patients with regard to ACPA risk factors (HLA-SE and smoking), the presence of IgM-RF and clinical parameters (UA to RA progression and joint destruction), but do not differ from anti-CCP-2-negative patients.

The fact that the current analysis made use of a single platform is a limitation. Nonetheless, the findings are in line with results obtained by other platforms [10, 24, 26]. Wagner et al. performed the most detailed analysis so far by using a bead-based array that included a larger number of citrullinated antigen (n = 16) in a small cohort of patients with RA, disease controls and healthy controls (n = 78) [10]. In this cohort, array positivity (defined as positive for more than one reactivity) was poorly RA-specific (50 %). Replication in a larger cohort of patients with RA and healthy controls without disease controls resulted in a high specificity of 95 %, supporting the importance of disease controls for multiplex validation studies. When array positivity was defined as positive for more than two reactivities, the specificity increased to 85 %. Likewise, when we determined specificity as positive for more than one epitope, the specificity also increased (from 54 % to 85 %). In our study, this is explained mostly by the exclusion of patients who are single-positive for Fibα36-50 or Fibβ563-583 that had poor discriminative capacity. Wagner et al. showed that eight out of the 16 antigens that they tested had no discriminative capacity, confirming the importance of a critical selection of applied antigens [10].

Wagner et al. found that at least 10 % of anti-CCP-2-negative RA patients were ACPA-positive. In this analysis, ACPA-positive was much more stringently defined (positive for more than two antigen reactivities with positive defined as mean + 3x the SD of the signal in healthy controls). Consistent with our findings, they also described that anti-CCP-2-negative RA patients who are positive for citrulline reactivities recognize a more restricted peptide repertoire [10]. Likewise, this observation was also confirmed in another study describing a surface plasmon resonance-based multiplex platform [24].

Multiplex assays are designed to investigate the presence of antibodies to a large number of targets in a high-throughput manner and with very small amounts of samples. The assay contains a large number of different peptides, which are derived from native proteins. It is not clear, however, whether antibodies to the peptides selected in the array in fact crossreact to native citrullinated proteins *in vivo*. It has been shown that antibodies to citrullinated type II collagen both bind joints *in vivo* and induce arthritis in mice, but the included peptide contains two citrullines that block many antibodies reactive with single citrullines present on type II collagen in cartilage [26, 27]. Thus, it is not clear whether any of the selected peptides attract antibodies that have functional relevance *in vivo*. On the other hand, this can also be said regarding the CCP-2 test since in this case the sequence is not publically known and therefore no arginine control can be used. Possibly the test contains several peptides that have been selected to give the highest specificity for RA. Thus, neither of the used tests gives any information on possible pathogenic or regulatory influence of the respective antibody reactivities.

In this study, we have focused on the clinical relevance of citrulline reactivities in anti-CCP-2-negative RA patients. To the best of our knowledge, only one other report has focused on the clinical relevance of citrulline reactivities in anti-CCP-2-negative patients. Pratt et al. studied the predictive value of reactivity toward citrullinated antigens in an ELISA platform with regard to UA to RA progression and found that none of the tested reactivities was as good as anti-CCP-2 in predicting progression. In line with our observations, reactivities to a set of citrullinated peptides in anti-CCP-2-negative UA patients were not associated with disease progression [28]. Although our findings are in line with results from other platforms, future work should focus on comparing citrulline reactivities in independent cohorts of anti-CCP-2-negative RA patients side by side on different platforms. To improve multiplex platforms, this work should be directed toward identifying those citrullinated peptides that have an additive value and are specific for RA or a specific phenotypical aspect such as the anti-SSA antibodies. Most importantly, such specified clinical characteristics should be linked with functional knowledge of the detected antibodies. These platforms need to include more relevant peptides and these platforms should be validated by using large cohorts of healthy controls, patients with RA and disease controls. In this regard, it would be important to compare the specificity of different peptides in multiple conformations (e.g., linear and cyclic). Future studies will also be useful to analyse the relationship of each reactivity with risk factors and prognosis and to investigate the functional role of these autoantibody specificities in the disease process. This could facilitate the incorporation of the most meaningful reactivities in future assays.

Our data indicate that the positive signals found in anti-CCP-2-negative sera using the current multiplex system should be interpreted with caution. The detected signals are dependent on the particular assays of peptides bound to solidphase, known to twist the conformation and increase variability and false-positive signals as compared with assaying protein-protein interactions. In addition, for most of the positive signals for detection of citrullinated peptides, the signal is just above the cutoff. False-positive signals may therefore contribute to the observation that citrulline reactivities in anti-CCP/MCV-negative RA patients are not associated with any of the known risk factors for ACPA-positive disease. Another limitation is that the peptide composition of the array is limited and the truly relevant citrullinated peptides or proteins are not present. This is especially relevant since the number of non-fibrinogen peptide-positive, anti-CCP-2-negative, multiplex-positive patients is low, making it impossible to analyse these reactivities. In fact, an increasing number of citrullinated peptides, not used in the present array, are reported to have potential pathogenic properties in the development of arthritis [27, 29].

## Conclusions

Our data indicate that residual reactivities to a wide range of citrullinated joint antigens as measured in the current multiplex system are unable to subdivide anti-CCP-2-negative RA patients into clinically different patient subsets. Nonetheless, it is clear that anti-CCP-2-negative RA patients are clinically heterogeneous and there is a need for biomarkers for disease phenotypes within diseases like RA but also across different diseases. This would require different studies in patient cohorts with detailed phenotypical characteristics which are selected in non-biased ways [30]. An improved multiplex assay could potentially accomplish this.

## Additional files

Additional file 1:
**Diagnosis of anti-CCP-2-negative non-RA patients.** Diagnoses of anti-CCP-2-negative non-RA patients (n = 135) as assessed by an experienced rheumatologist after 1 year of follow-up stratified for multiplex positivity. *CCP* cyclic citrullinated peptide, *RA* rheumatoid arthritis. (PDF 14 kb) Additional file 2:
**Baseline characteristics of anti-CCP-2-negative RA** **patients.** Baseline characteristics of anti-CCP-2-negative RA patients (n = 279) that are multiplex-negative or -positive. *CCP* cyclic citrullinated peptide, *RA* rheumatoid arthritis. (PDF 40 kb) Additional file 3:
**Peptides recognized by anti-CCP-2-negative EAC patients.** Peptides recognized by anti-CCP-2-negative EAC patients who are positive in the multiplex assay for a single epitope (n = 143). *CCP* cyclic citrullinated peptide, *EAC* Leiden Early Arthritis Clinic, *RA* rheumatoid arthritis. (PDF 24 kb) Additional file 4:
**Citrulline reactivities to Fibα36-50 and Fibβ563-583.** **a** Citrulline reactivities toward Fibβ563-583 in anti-CCP-2-positive (n = 285) and anti-CCP-2-negative (n = 279) RA patients and non-RA patients (n = 149) in the EAC. **b** Citrulline reactivities in arbitrary units (AUs) toward Fibβ563-583 in anti-CCP-2-positive (n = 174) and anti-CCP-2-negative (n = 92) RA patients in the IMPROVED cohort. **c** Citrulline reactivities toward Fibα36-50 in anti-CCP-2-positive (n = 285) and anti-CCP-2-negative (n = 279) RA patients and non-RA patients (n = 149) in the EAC. **d** Citrulline reactivities toward Fibα36-50 in anti-CCP-2-positive (n = 174) and anti-CCP-2-negative (n = 92) RA patients in the IMPROVED cohort. The dotted line indicates the cutoff. Reactivities are plotted in AUs. Each dot indicates a unique individual. *CCP* cyclic citrullinated peptide, *EAC* Leiden Early Arthritis Clinic, *IMPROVED*, *RA* rheumatoid arthritis. (PDF 397 kb)

## Abbreviations

ACPA: Anti-citrullinated protein antibody
ACR: American College of Rheumatology
CCP: Cyclic citrullinated peptide
CI: Confidence interval
EAC: Leiden Early Arthritis Clinic
ELISA: Enzyme-linked immunosorbent assay
IgM-RF: IgM-rheumatoid factor
IMPROVED: Induction therapy with Methotrexate and Prednisone in Rheumatoid Or Very Early arthritic Disease
ISAC: Immuno Solid-phase Alergen Chip
LR: Likelihood ratio
MCV: Mutated citrullinated vimentin
OR: Odds ratio
RA: Rheumatoid arthritis
RF: Rheumatoid factor
SD: Standard deviation
SE: Shared epitope
SHS: Sharp-van der Heijde score
SSA: Sjögren’s syndrome-related antigen A
UA: Undifferentiated arthritis
