# Supplementary material for: An investigation of the added value of an ACPA multiplex assay in an early rheumatoid arthritis setting
Source: Arthritis Res Ther. 2015 Oct 5;17:276. doi: 10.1186/s13075-015-0786-z (PMC4595184; doi:10.1186/s13075-015-0786-z)
Supplement: Additional file 1: — Diagnosis of anti-CCP-2-negative non-RA patients. Diagnoses of anti-CCP-2-negative non-RA patients (n = 135) as assessed by an experienced rheumatologist after 1 year of follow-up stratified for multiplex positivity. CCP cyclic citrullinated peptide, RA rheumatoid arthritis. (PDF 14 kb) [file 13075_2015_786_MOESM1_ESM.pdf]

Additional file 1

**Group**	**Multiplex-negative**	**Multiplex-positive**	**Total**
Reactive arthritis	6 (6%)	4 (11%)	10 (7%)
Gout	16 (16%)	6 (16%)	22 (16%)
Pseudogout	1 (1%)	0 (0%)	1 (1%)
Spondyloarthritis including psoriatric arthritis	45 (46%)	13 (35%)	58 (43%)
Osteoarthritis	15 (15%)	6 (16%	21 (16%)
Lyme arthritis	1 (1%)	0 (0%)	1 (1%)
Paramalignant arthritis	0 (0%)	4 (11%)	4 (3%)
Sarcoidosis	0 (0%)	1 (3%)	1 (1%)
Mixed connective tissue disease, vasculitis	1 (1%)	0 (0%)	1 (1%)
Other systemic autoimmune diseases	5 (5%)	0 (0%)	5 (4%)
Remitting seronegative symmetrical synovitis with pitting edema	8 (8%)	2 (8%)	10 (7%)
Rest	0 (0%)	1 (3%)	1 (1%)
**Total**	**98 (100%)**	**37 (100%)**	**135 (100%)**
