# Supplementary material for: An investigation of the added value of an ACPA multiplex assay in an early rheumatoid arthritis setting
Source: Arthritis Res Ther. 2015 Oct 5;17:276. doi: 10.1186/s13075-015-0786-z (PMC4595184; doi:10.1186/s13075-015-0786-z)
Supplement: Additional file 2: — Baseline characteristics of anti-CCP-2-negative RA patients. Baseline characteristics of anti-CCP-2-negative RA patients (n = 279) that are multiplex-negative or -positive. CCP cyclic citrullinated peptide, RA rheumatoid arthritis. (PDF 40 kb) [file 13075_2015_786_MOESM2_ESM.pdf]

Additional file 2

Patient Characteristic	Multiplex-negative n=193	Multiplex-positive n=86	P-value
**Anti-CCP2 titer in AU/ml, mean (SD)**	13 (9)	12 (10)	0.53
**median (IQR)**	20 (3-21)	20 (1-20)	0.73
**Age in yrs, mean (SD)**	60 (17)	59 (17)	0.67
**Female gender, n (%)**	47 (55)	131 (68)	0.04
**HAQ, median (IQR)**	1.0 (0.6-1.6)	1.0 (0.6-1.6)	0.76
**mean (SD)**	1.1 (0.7)	1.1 (0.7)	0.92
**CRP in mg/ml, mean (SD)**	27 (30)	35 (42)	0.72
**median (IQR)**	15 (6-37)	23 (6-48)	0.46
**SHS, mean (SD)**	9 (11)	8 (9)	0.36
**median (IQR)**	6 (2-12)	4 (2-11)	0.19
**ESR in mm/h, mean (SD)**	35 (23)	33 (27)	0.54
**Symptom duration at baseline in months, mean (SD)**	6 (12)	5 (9)	0.58
