# Supplementary material for: An investigation of the added value of an ACPA multiplex assay in an early rheumatoid arthritis setting
Source: Arthritis Res Ther. 2015 Oct 5;17:276. doi: 10.1186/s13075-015-0786-z (PMC4595184; doi:10.1186/s13075-015-0786-z)
Supplement: Additional file 3: — Peptides recognized by anti-CCP-2-negative EAC patients. Peptides recognized by anti-CCP-2-negative EAC patients who are positive in the multiplex assay for a single epitope (n = 143). CCP cyclic citrullinated peptide, EAC Leiden Early Arthritis Clinic, RA rheumatoid arthritis. (PDF 24 kb) [file 13075_2015_786_MOESM3_ESM.pdf]

Additional file 3

Group	Number	Percentage
CEP1	0	0%
Vim2-17	0	0%
Fibβ36-52	1	1%
Fibα621-635	0	0%
Fibβ680-600	0	0%
CCP1	1	1%
C1	4	3%
Vim60-75	6	4%
Fibβ60-74	9	6%
Fibβ563-583	49	34%
Fibα36-50	73	51%
**Total**	**143**	**100%**
