# Supplementary figures and images for: An investigation of the added value of an ACPA multiplex assay in an early rheumatoid arthritis setting
Source: Arthritis Res Ther. 2015 Oct 5;17:276. doi: 10.1186/s13075-015-0786-z (PMC4595184; doi:10.1186/s13075-015-0786-z)

**Additonal file 4**

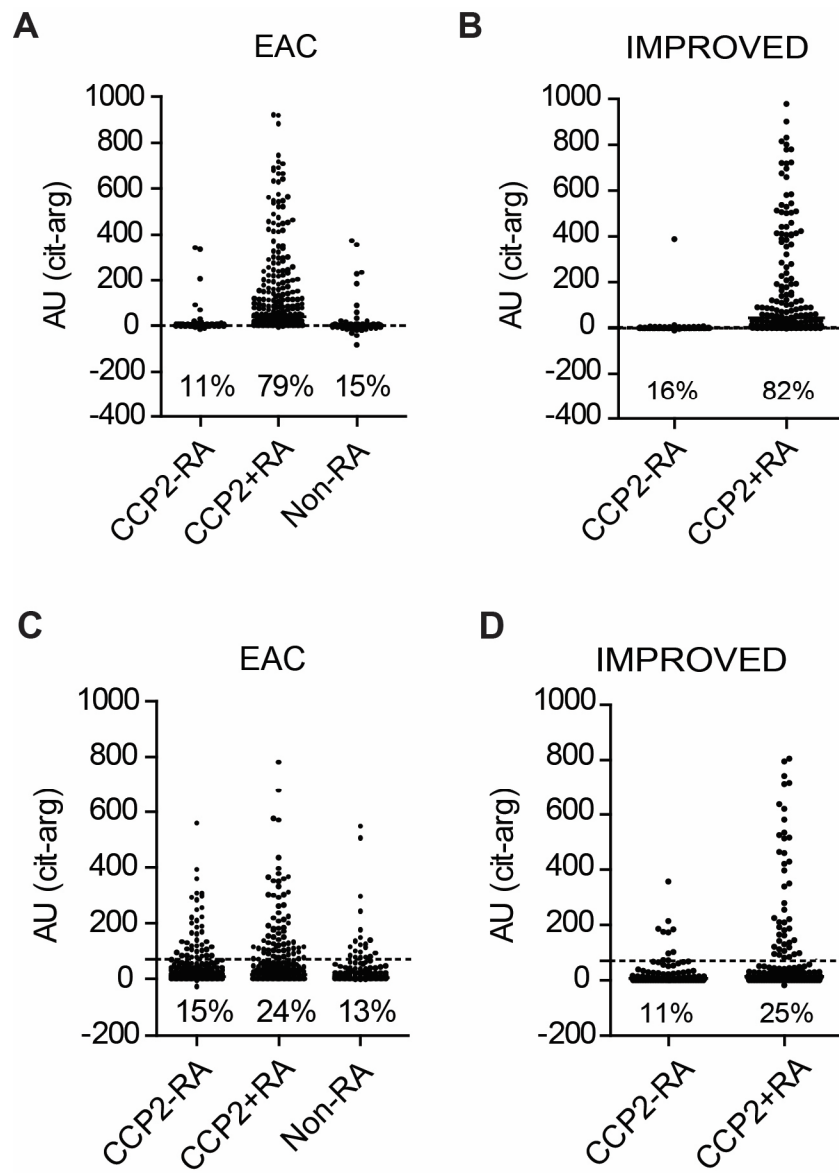

Supplement: Additional file 4: — Citrulline reactivities to Fibα36-50 and Fibβ563-583. a Citrulline reactivities toward Fibβ563-583 in anti-CCP-2-positive (n = 285) and anti-CCP-2-negative (n = 279) RA patients and non-RA patients (n = 149) in the EAC. b Citrulline reactivities in arbitrary units (AUs) toward Fibβ563-583 in anti-CCP-2-positive (n = 174) and anti-CCP-2-negative (n = 92) RA patients in the IMPROVED cohort. c Citrulline reactivities toward Fibα36-50 in anti-CCP-2-positive (n = 285) and anti-CCP-2-negative (n = 279) RA patients and non-RA patients (n = 149) in the EAC. d Citrulline reactivities toward Fibα36-50 in anti-CCP-2-positive (n = 174) and anti-CCP-2-negative (n = 92) RA patients in the IMPROVED cohort. The dotted line indicates the cutoff. Reactivities are plotted in AUs. Each dot indicates a unique individual. CCP cyclic citrullinated peptide, EAC Leiden Early Arthritis Clinic, IMPROVED, RA rheumatoid arthritis. (PDF 397 kb) [file 13075_2015_786_MOESM4_ESM.pdf]
